# Numerical Simulation and Clinical Implications of Stenosis in Coronary Blood Flow

**DOI:** 10.1155/2014/514729

**Published:** 2014-06-02

**Authors:** Jun-Mei Zhang, Liang Zhong, Tong Luo, Yunlong Huo, Swee Yaw Tan, Aaron Sung Lung Wong, Boyang Su, Min Wan, Xiaodan Zhao, Ghassan S. Kassab, Heow Pueh Lee, Boo Cheong Khoo, Chang-Wei Kang, Te Ba, Ru San Tan

**Affiliations:** ^1^National Heart Center Singapore, 5 Hospital Drive, Singapore 169609; ^2^Duke-NUS Graduate Medical School Singapore, 8 College Road, Singapore 169857; ^3^Department of Biomedical Engineering, Indiana University-Purdue University Indianapolis, Indianapolis, IN 46202, USA; ^4^Department of Mechanics and Engineering Science, College of Engineering, Peking University, Beijing 100871, China; ^5^Department of Mechanical Engineering, National University of Singapore, 1 Engineering Drive 2, Singapore 117576; ^6^Fluid Dynamics Department, Institute of High Performance Computing, 1 Fusionopolis Way, No. 16-16 Connexis North, Singapore 138632

## Abstract

Fractional flow reserve (FFR) is the gold standard to guide coronary interventions. However it can only be obtained via invasive angiography. The objective of this study is to propose a noninvasive method to determine FFR_CT_ by combining computed tomography angiographic (CTA) images and computational fluid dynamics (CFD) technique. Utilizing the method, this study explored the effects of diameter stenosis (DS), stenosis length, and location on FFR_CT_. The baseline left anterior descending (LAD) model was reconstructed from CTA of a healthy porcine heart. A series of models were created by adding an idealized stenosis (with DS from 45% to 75%, stenosis length from 4 mm to 16 mm, and at 4 locations separately). Through numerical simulations, it was found that FFR_CT_ decreased (from 0.89 to 0.74), when DS increased (from 45% to 75%). Similarly, FFR_CT_ decreased with the increase of stenosis length and the stenosis located at proximal position had lower FFR_CT_ than that at distal position. These findings are consistent with clinical observations. Applying the same method on two patients' CTA images yielded FFR_CT_ close to the FFR values obtained via invasive angiography. The proposed noninvasive computation of FFR_CT_ is promising for clinical diagnosis of CAD.

## 1. Introduction

As the most common type of cardiovascular disease, coronary artery disease (CAD) is caused by the build-up of plaques on the endothelial walls of coronary arteries, which leads to a reduction in arteries cross-sectional area and blood supply to the myocardium [1]. Pathology studies of CAD have revealed that coronary stenosis is the predominant factor leading to cardiovascular-related events such as myocardial infarction (MI), stroke, and unstable angina. Therefore it is vital to physiologically characterize and quantify functional coronary stenosis.

The rapid development of noninvasive imaging technologies, such as computed tomography angiography (CTA) and magnetic resonance imaging (MRI), has proven valuable to characterize the anatomic severity of CAD with fair cost and less complication. Diameter stenosis (DS) is commonly applied to quantify the anatomic severity of CAD. It expresses the ratio of the lumen diameter at a stenotic region over that of a “normal” segment. However, DS cannot characterize hemodynamic functional significance of coronary stenosis on myocardial blood supply. Among the stenoses discriminated with the threshold of 50% for DS, fewer than half are ischemic [2].

In terms of hemodynamic indexes to quantify the functional significance of coronary stenosis, fractional flow reserve (FFR) is the gold standard to guide coronary interventions [3–6] because of its significantly fewer follow-up coronary events over DS. FFR is calculated as a ratio of the pressure distal versus pressure proximal to a stenosis following vasodilation. Revascularization is commonly recommended when the coronary stenosis leads to FFR ≤ 0.80. However, FFR can only be measured via invasive coronary catheterization, which may result in higher medical cost and complications [4]. This is not ideal, because only one-third of the intermediate stenosis (DS = 40–70%) is classified as functional significant with FFR ≤ 0.80 [5].

In view of the abundant hemodynamic information provided by computational fluid dynamics (CFD) simulations, considerable insights have been gained on understanding the physiology of CAD through CFD studies [7]. Tremendous progress has been made in applying the CFD method to simulate the blood flow in patient-specific coronary artery models for elucidating the role of hemodynamics in CAD development and progression [8–10].

As proper boundary conditions are necessary for CFD simulations of CAD, both prescribed profiles [11, 12] and sophisticated reduced-ordered models [13–15] have been attempted. For the latter, the downstream vasculature is represented by flow-dependent formulation. For example, the static pressure at the outlet may be calculated from the corresponding flow rate and resistance. Since resistance, compliance, or impedance values of the downstream vasculature are assumed to be decided by their own anatomy rather than by the upstream stenosis, these parameters obtained from the normal vessel (without stenosis) can be applied to the diseased vessel [15].

With the development of CFD techniques, numerous CFD studies have been coupled with clinical imaging techniques to provide detailed hemodynamic information and link CAD with vortices [16], wall shear stress (WSS) [12, 17], oscillatory shear index (OSI) [18], and so forth. However, the application of CFD method to predict clinical diagnosis indexes is still limited. A landmark study was carried out by HeartFlow Company [19–22] to derive noninvasive FFR_CT_ from CT images by applying sophisticated reduced-ordered models as the boundary conditions for transient numerical simulations. Because the calculation of FFR is based on the time-averaged pressure over several cardiac cycles in clinics [23], this study explored a method to combine CT images and steady flow simulation for calculating noninvasive FFR_CT_ with lower computational cost. The method is firstly applied on a left anterior descending (LAD) model reconstructed from CTA of a healthy porcine heart and then tested on two patient-specific left coronary artery trees reconstructed from patients' CTA images to examine whether the approach can be applied for noninvasively assessing the hemodynamic significance of coronary stenosis in clinics.

As the anatomical severity of CAD is believed to be related not only to DS but also to the location and length of the stenosis, which can affect the hemodynamics [24], a series of models were created by adding an idealized stenosis (with DS from 45% to 75%, stenosis length from 4 mm to 16 mm, and at 4 locations separately) on the baseline porcine LAD model. In this manner, the effects of DS, stenosis length, and location (proximal to distal) on FFR_CT_ are investigated. The results may be useful to aid the clinician in making the decision of revascularization.

This paper is organized as follows: the detailed computational methodologies were described in Section 2. In Section 3, the detailed hemodynamic information (flow patterns, flow rate, pressure, and FFR_CT_ distributions) for a series of stenosed porcine LAD models was provided to explore the effect of DS, stenosis length, and location on FFR_CT_. The results of two case studies were also presented to demonstrate the applicability of the current methodologies on clinical diagnosis of human CAD. Finally, the conclusions were drawn in Section 4.

## 2. Computational Methodologies

**Figure 1:**
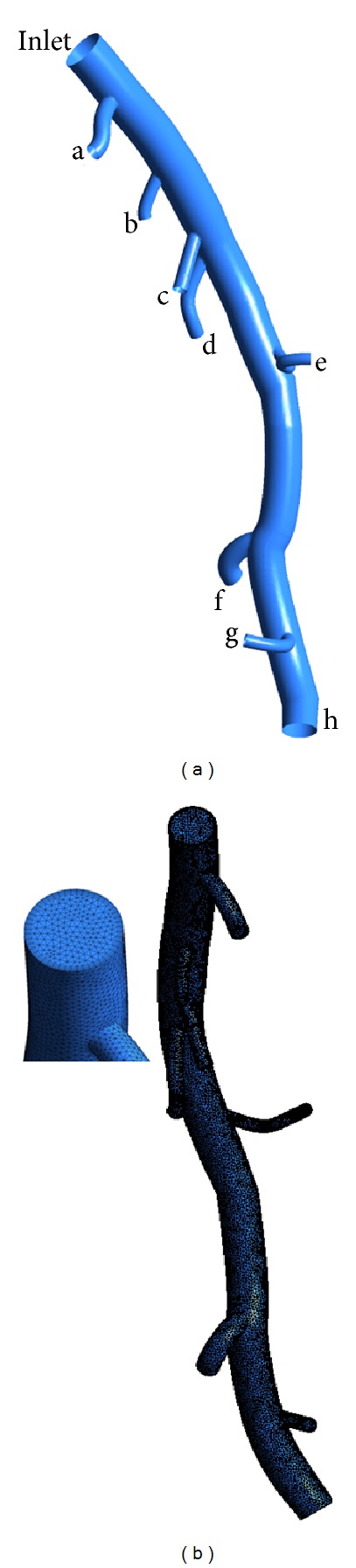
(a) Baseline LAD model reconstructed from CT and (b) the generated meshes for the baseline model with enlarged view.

**Figure 2:**
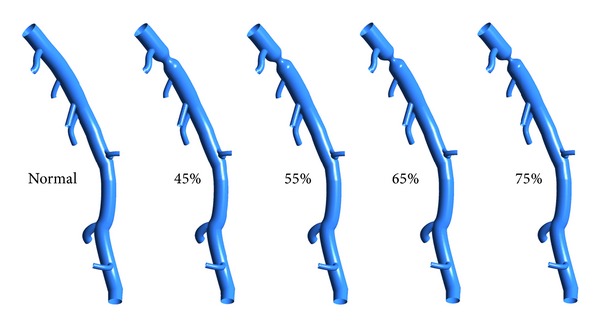
Baseline and stenosed LAD models with the 4 mm length stenoses located at the same location (Location A) with DS of 45%, 55%, 65%, and 75% separately.

**Figure 3:**
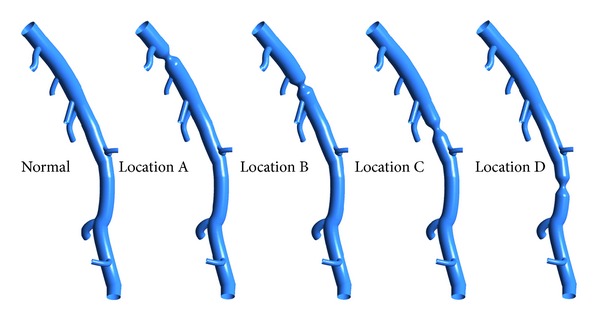
Baseline and stenosed LAD models with the 4 mm length stenoses located at A, B, C, and D separately (having the DS of 55%).

**Figure 4:**
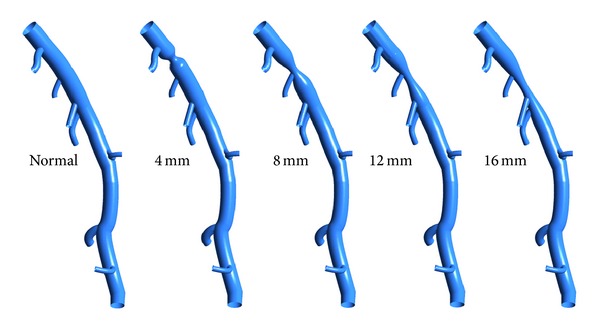
Baseline and stenosed LAD models with 55% DS located downstream the branch “a” with the stenosis lengths of 4 mm, 8 mm, 12 mm, and 16 mm separately.

In this study, a left anterior descending (LAD) model was used as the baseline model, which was reconstructed from CTA of a healthy porcine heart. The detailed description on animal preparation, CTA, and image processing can be found in [25]. As shown in Figure 1(a), the baseline model embodied the LAD main trunk (≥2 mm in diameter) and 7 primary branches (“a”–”g” in Figure 1) (≥1 mm in diameter). To investigate the effect of DS, the trunk between side branches “a” and “b” was narrowed to represent stenosed model with DS of 45%, 55%, 65%, and 75% separately as shown in Figure 2. All of these stenoses were located at the same location (Location A) with the same stenosis length of 4 mm. To investigate the effect of stenosis location, a 4 mm length stenosis with DS of 55% was created at 4 locations (Location “A” to “D”) from proximal to distal LAD trunk, as shown in Figure 3. Another series of models were constructed with a 55% stenosis located downstream the branch “a” and having the stenosis lengths of 4 mm, 8 mm, 12 mm, and 16 mm separately, as shown in Figure 4.

After generating these LAD models with SpaceClaim, their computational domains were discretized with commercial software ANSYS workbench. Meshes near the branch junctions, at the stenosis and near the walls, were refined for adequate resolution of flow in the boundary layers (as shown in Figure 1(b)). After mesh dependency test conducted on the baseline model, a total of about 0.5 million volume cells were found to be adequate, as further grid refinement led to less than 1% relative error in the maximum velocity. The same setting for mesh generation was applied for all other LAD models.

In order to simulate the blood flow in normal and diseased LAD models, the continuity (1) and Navier-Stokes (2) equations were solved by FLUENT using finite volume approximation:

(1) $$\begin{array}{c} \frac{\partial u_{j}}{\partial x_{j}}=0 \end{array}$$

(2) $$\begin{array}{c} \frac{\partial \left(\rho u_{j}u_{i}\right)}{\partial x_{j}}=-\frac{\partial P}{\partial x_{i}}+\frac{\partial }{\partial x_{j}}\left(\mu \left(\frac{\partial u_{i}}{\partial x_{j}}+\frac{\partial u_{j}}{\partial x_{i}}\right)\right), \end{array}$$

Here *x*_*j*_ and *u*_*j*_ (or *u*_*i*_) were the location in Cartesian coordinate and the Cartesian component of velocity, respectively. *P* represented the static pressure. *ρ* and *μ* were density and dynamic viscosity of the fluid, which were set as 1060 kg/m^3^ and 4.5 × 10^−3^ Pa·s, respectively, to mimic the blood properties in large epicardial arteries [25, 26].

Proper boundary conditions are required for CFD simulations to closely mimic the real physiological condition [13]. In general, the total pressure was specified at the inlet of each model, and resistance boundary condition was assigned to every coronary outlet to enforce a relationship between pressure and flow.

According to the pulsatile pressure and flow velocity waveforms measured in vivo at the inlet of the porcine LAD [25], the time-averaged inflow rate and pressure were 66.75 mL/min and 69.54 mmHg, respectively.

In order to derive the resistance values of downstream vasculatures, the steady-state simulations under two sets of boundary conditions were carried out on the baseline model prior to the simulation on stenosed LAD models. One of the simulations represents the peak phase of systole, when the inlet pressure is at the maximum [25]. The other simulation represents the peak diastole phase with minimum pressure at the inlet. For both simulations, the flow rate distributions between the primary branches were believed to obey the generalized Murray's law [27], as it was the baseline LAD, which represents normal (healthy) coronary arteries without stenosis. Based on these two steady-state flow simulations, the pressure and flow rate information at the outlets of primary branches could be obtained. Accordingly the resistance of the vasculature downstream each primary branch was obtained via

(3) $$\begin{array}{c} R_{i}=\frac{P_{i}-P_{0,i}}{Q_{i}}, \end{array}$$

where *P*_*i*_ and *Q*_*i*_ represent the pressure and flow rate at the *i*th outlet, respectively. Here, *R*_*i*_ is the resistance of the downstream vasculature and *P*_0,*i*_ is the back pressure at *i*th outlet.

In clinics, FFR measurement is done following vasodilation, which leads to the decrease of coronary resistance. To resemble this physiological situation, the resistance of downstream vasculature of each primary branch was assumed to be reduced to 0.21 times of its resting value, which was within the physiological range measured by Wilson et al. [24]. Accordingly the total pressure at the inlet was assumed to be 69.87 mmHg for all the porcine LAD models with the consideration of flow rate increment following vasodilation. Because the epicardial stenosis severity was found to be not affecting the minimal microvascular resistance [28], the resistance of downstream vascular for a stenosed LAD model was assumed to be the same as that of the baseline model [22].

User defined function (UDF) was compiled to supply the resistance boundary conditions at the outlets. In this manner, the downstream vasculature of the primary branches was coupled to the LAD model, and the static pressure at each outlet was solved iteratively. To ensure smooth convergence, the pressure gradient calculated at the outlet was attenuated several times to couple iterative underrelaxation-based resistance boundary conditions with those outlets; namely, an implicit algorithm was applied to the outlet with an underrelaxation factor of *ω* given as

(4) $$\begin{array}{c} P_{i}^{n+1}=P_{i}^{n}+\omega \left(R_{i}Q_{i}^{n+1}+P_{o,i}-P_{i}^{n}\right). \end{array}$$

In addition, no-slip boundary condition was applied at the wall, as all these LAD models were assumed to be stationary and rigid. All the computations were executed in a Dell T7500 workstation and it took around half hour computational time for one case.

## 3. Results and Discussion

**Figure 5:**
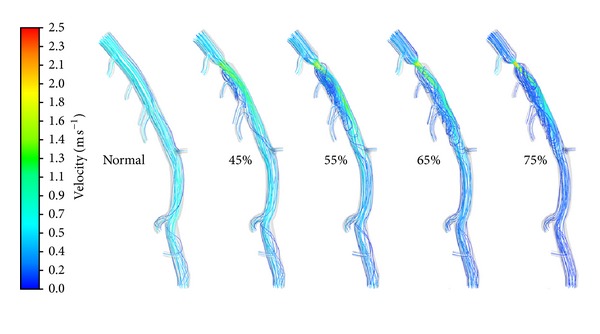
Streamline distributions on the baseline and stenosed LAD models with the 4 mm length stenoses located at the same location (Location A) with DS of 45%, 55%, 65%, and 75% separately.

To elucidate the role of stenosis on hemodynamics of coronary artery, Figure 5 shows the streamline distributions in the normal and stenosed LAD models with DS increasing from 45% to 75% at interval of 10%. When the LAD was free of stenosis, part of the inflow diverted in the primary branches along its way downstream. Most of the blood flowed through the LAD main trunk smoothly, with streamlines parallel to the walls. In other words, there was no obvious presence of vortex in the normal LAD model.

Once there was a stenosis in the LAD trunk, the streamlines diverted into the center of the LAD main trunk due to the shrinking of the cross-sectional area from the beginning to the throat of the stenosis region. Downstream the throat of the stenosis, the cross-sectional area of the LAD main trunk increased gradually. Due to the inertia momentum, the fluid moved downstream with slight divergence of the streamlines. Hence flow recirculation region was formed near the wall downstream the stenosis, which was filled by vortices and reverse flow derived from the downstream streamlines as shown in Figure 5. With the increase of DS, the throat diameter became smaller leading to more significant divergence of the streamlines and larger flow recirculation in size downstream the stenosis. These vortices and flow recirculation were suspected to promote thrombus formation and potentially myocardial infarction [16].

**Figure 6:**
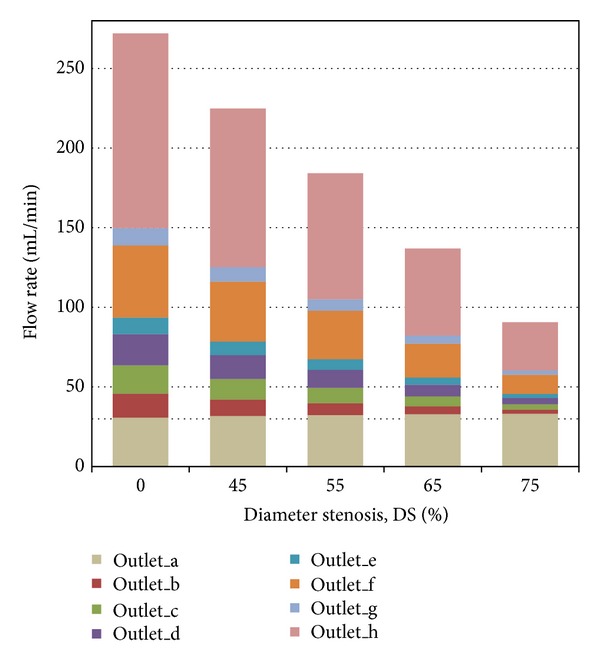
Flow rate distributions for the baseline and stenosed LAD models with the 4 mm length stenoses located at the same location (Location A) with DS of 45%, 55%, 65%, and 75% separately.

An interesting phenomenon was found for the flow rate distribution among the primary branches. With the increase of DS, less blood flowed downstream to perfuse the myocardium downstream the first primary branch (labeled as “a” in Figure 1). This is because, for this series of models, the stenosis was located at the main trunk downstream the first primary branch. The vortices and flow recirculation developed downstream the stenosis result in energy diffusion and dissipation, which makes it difficult to perfuse the blood downstream. Hence less blood was distributed to the regions downstream the stenosis. However, there was a slight increase of blood flow to the first primary branch, with the increase of DS as shown in Figure 6. Similar phenomenon is named as “branch steal” by Gould et al. [29] to describe the situation when a nonstenotic branch between proximal and distal stenoses shunts flow away from the stenotic parallel daughter branch to an extent depending on their relative size and severities of the 2 stenoses, which leads to less flow in the stenotic daughter branch. It was also found that the sum of the total blood flow through the outlets reduced with the increase of DS, which implied that stenosis led to a reduction in blood supply to the myocardium. This was the most important physiological complication of coronary stenosis.

**Figure 7:**
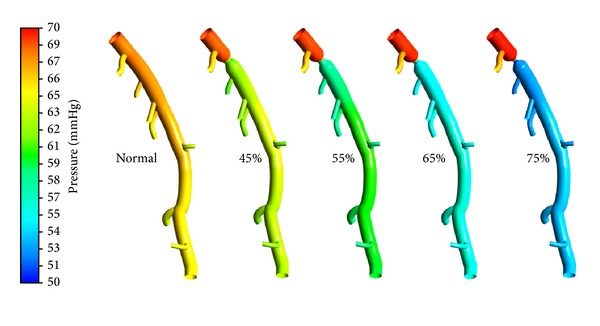
Pressure distributions on the baseline and stenosed LAD models with the 4 mm length stenoses located at the same location (Location A) with DS of 45%, 55%, 65%, and 75% separately.

To quantify the hemodynamic significance of stenosis, Figure 7 shows the pressure distribution for different models. For normal LAD, the pressure drop along the main trunk was mainly due to the viscous force, which was less than 3.3 mmHg. Due to the diffusion and dissipation of the recirculation vortices formed downstream the stenosis, the pressure therein decreased much more. With the increase of DS, pressure downstream the stenosis became much lower as shown in Figure 7.

**Figure 8:**
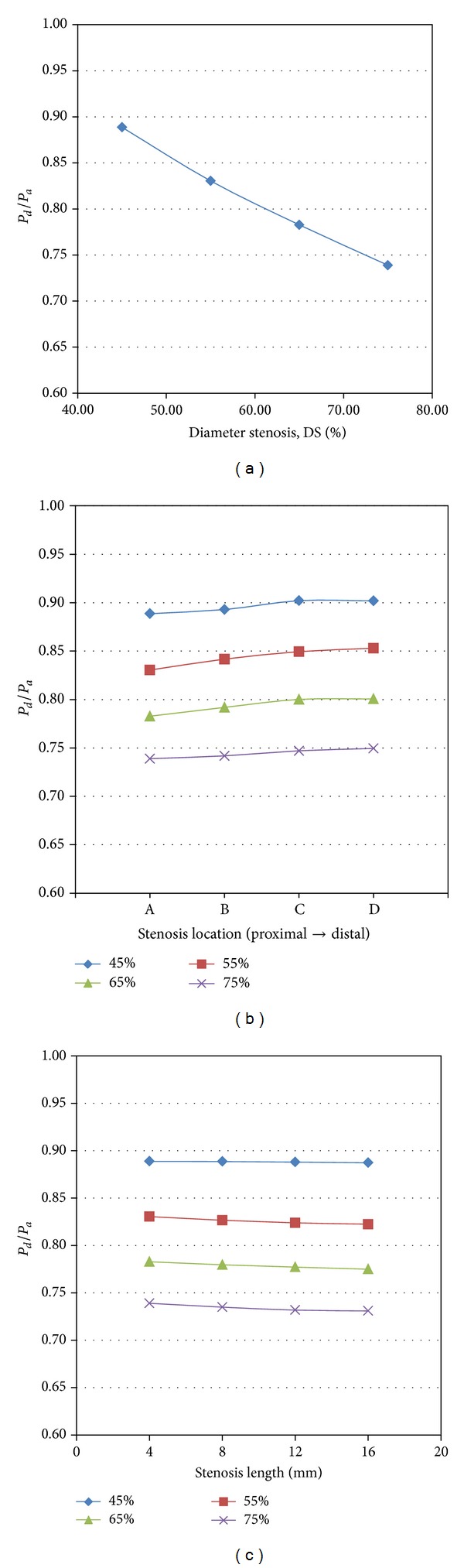
Effect of (a) diameter stenosis, (b) stenosis location, and (c) length on FFR_CT_.

To resemble FFR values measured via invasive angiography, FFR_CT_ is calculated as *P*_*d*_/*P*_*a*_. Here *P*_*d*_ and *P*_*a*_ represent the pressure distal and proximal to the stenosis, respectively. As shown in Figure 8(a), FFR_CT_ decreased from 0.89 to 0.74, when DS increased from 45% to 75%. As revascularization is commonly recommended when coronary stenosis leads to FFR ≤ 0.8 in clinical applications [5], the LAD with DS ≥ 60% was suggested to receive revascularization procedures according to Figure 8(a). This is in line with the clinical findings that the diagnostic performance of CTA can be improved by rating coronary stenosis as significant with the threshold of 60% rather than 50% [30].

To assess the effect of stenosis location and length on the hemodynamic severity of CAD, Figures 8(b) and 8(c) show the relationship between stenosis location and length with FFR_CT_, respectively. Comparing with the stenosis located more distally, those stenoses located at proximal portion led to lower *P*_*d*_/*P*_*a*_, namely, FFR_CT_. Therefore the stenosis located at proximal portion resulted in more significant reduction in blood supply to the myocardium, which was suspected to promote the further accumulation of plaques. This result is consistent with the clinical findings that the adverse events of coronary artery disease occurred most frequently in a proximal position [31].

In addition, the FFR_CT_ values decreased with the increase of stenosis length, which implies higher fractional losses over the stenosis. However the effect of lesion length on hemodynamics is not significant as shown in Figure 8(c). Similar finding was reported by Wilson et al. [24]. However it is worth noting that fractional losses at longer stenosis may depend on the ruggedness of the surface and local geometry of the stenosis in real life. Hence simulation on the realistic coronary artery tree is critically important.

**Figure 9:**
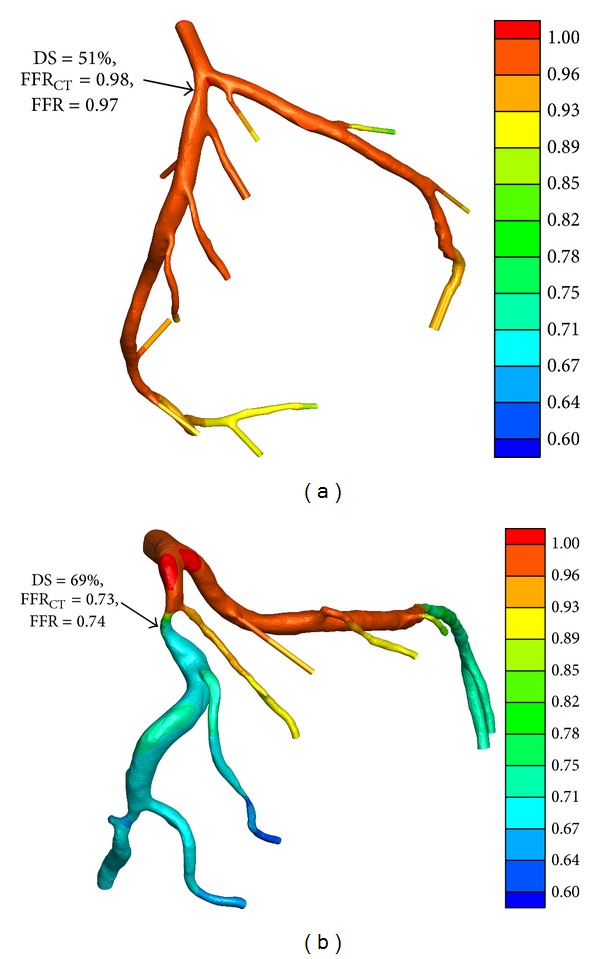
Distributions of FFR_CT_ based on numerical simulations on patient-specific left coronary trees reconstructed from two patients' CTA images. (a) The patient has a moderate stenosis (DS = 51%) at proximal LAD, whose FFR is 0.97 via invasive angiography and its FFR_CT_ is 0.98 predicted by CFD. (b) The patient has a moderate stenosis (DS = 69%) at mid LAD, whose FFR is 0.73 via invasive angiography and its FFR_CT_ is 0.74 predicted by CFD.

Therefore, we have also attempted to apply the same CFD methodologies on the patients' CTA images. The detailed information on image reconstruction and numerical simulation was presented in [32, 33]. Figures 9(a) and 9(b) show the predicted FFR_CT_ on the patient-specific left coronary artery trees reconstructed from two patients' CTA images, respectively. The first patient has a moderate stenosis at proximal LAD, which is not ischemia-causing, as its FFR was 0.97 measured via invasive angiography. Its FFR_CT_ predicted by CFD was 0.98. The other patient has an ischemia-causing moderate stenosis at mid LAD, whose FFR was less than 0.8, that is, at 0.73, and its FFF_CT_ was found to be 0.74. These two examples demonstrated that the noninvasive methodologies presented in this study were applicable to assess the hemodynamic significance of stenosis in clinics.

## 4. Conclusions

In recent years, tremendous progress has been made on invasive and noninvasive medical imaging techniques to diagnose the anatomical and hemodynamic significance of coronary stenosis. Although FFR is the gold standard to diagnose the hemodynamic significance of the CAD, it can only be obtained via invasive coronary angiography. Combining CTA with CFD methods, the flow pattern and pressure distributions can be predicted for a baseline LAD model reconstructed from the CTA of a healthy porcine heart.

By adding stenosis on this baseline model, the effects of DS, stenosis location, and length on the hemodynamics of stenosed coronary artery were explored. It was found that the flow recirculation vortices were formed downstream the throat of stenosis due to the divergence of the streamline, which led to the decrease of flow rate and pressure downstream the stenosis due to energy diffusion and dissipation. The phenomenon of “branch steal” was observed, which was previously reported by Gould et al. [29] when a nonstenotic branch between proximal and distal stenoses shunts flow away from the stenotic parallel daughter branch [28].

With the increase of DS, the flow recirculation region increased in size, which led to lower pressure and FFR_CT_ downstream stenosis. Using a threshold of 0.8, the LAD with DS ≥ 60% was suggested to receive revascularization procedure rather than DS ≥ 50%, which is in line with the clinical finding [30].

In addition, the stenosis location and length were found to affect the hemodynamics in coronary artery trees. Lower FFR_CT_ was associated with the stenosis located in the proximal position and/or with longer stenosis length. This was in line with the clinical observations [24, 31].

Further application of the methodologies on two patient-specific left coronary artery trees reconstructed from CTA images demonstrated that the methodology utilized in this study was promising in facilitating the patient-specific noninvasive diagnosis of hemodynamic significance of coronary stenosis with affordable computational cost. Currently, the computation time was half hour for one case study with a Dell T7500 workstation, which is much longer than an invasive clinical setting, but it is acceptable for noninvasive diagnostics. If a more powerful workstation with larger number of compute nodes is applied, the computation time can be much less than 30 minutes.

Although efforts have been made to mimic the physiological condition as close as possible, there are several possible sources of limitation inherent in the assumptions made in the present study. First, the determination of downstream vascular resistance is argumentative. Intracoronary resistance has been reported to be fluctuating in a phasic pattern, even after administration of vasodilation medicine [23, 34, 35], due to the interaction between the myocardium and microvasculature during systole and diastole. However a mean resistance value was estimated (by iterative approximation at two time points) in this study. To justify the effect of resistance value on FFR_CT_, a series of simulations were carried out on one model. It was found that the 24–71% variations of resistance values only led to less than 2.4% differences of FFR_CT_. Therefore the error in the estimated downstream vascular resistance may only result in limited effect on the results of FFR_CT_. In addition, the method in this study does not consider the circumference with microvascular disease, which is an interesting topic to be explored in the future.

Secondly, the compliance effect of the vessel walls was ignored in this study, as compliance was reported to be less important in affecting the hemodynamics of the coronary arteries [36]. Thirdly, the blood flow was assumed to be laminar with Newtonian fluid. Since the vessels investigated in this study had a diameter greater than 1 mm and the blood flow in the elderly was generally correlated with the low hematocrit, the assumption of Newtonian fluid can be justified. In addition, as the peak Reynolds number in coronary arteries was lower than the critical value proposed by Peacock et al. [37], the flow can be assumed to be laminar in this study.

Last but not least, the impact of errors in image processing on the simulation results is worth exploring in the future. Current clinical imaging processing techniques have difficulty in reconstructing accurate 3D models for small vessels (e.g., with a diameter less than 0.5 mm) owing to the limitation of the imaging resolution. With the development of medical imaging techniques, the breakthrough in this area should be achievable in the near future.

## Abbreviations

CAD: Coronary artery disease
CFD: Computational fluid dynamics
CT: Computed tomography
DS: Diameter stenosis
FFR: Fractional flow reserve
LAD: Left anterior descending
MI: Myocardial infarction
OSI: Oscillatory shear index
WSS: Wall shear stress.
